# Regulation of Macrophage, Dendritic Cell, and Microglial Phenotype and Function by the SOCS Proteins

**DOI:** 10.3389/fimmu.2015.00549

**Published:** 2015-10-27

**Authors:** Sarah M. McCormick, Nicola M. Heller

**Affiliations:** ^1^Anesthesiology and Critical Care Medicine, The Johns Hopkins University, Baltimore, MD, USA; ^2^Allergy and Clinical Immunology, The Johns Hopkins University, Baltimore, MD, USA

**Keywords:** macrophage, suppressor of cytokine signaling proteins, IL-4 and IL-13, dendritic cells, macrophages, differentiation, M1 macrophage, M2 macrophages

## Abstract

Macrophages are innate immune cells of dynamic phenotype that rapidly respond to external stimuli in the microenvironment by altering their phenotype to respond to and to direct the immune response. The ability to dynamically change phenotype must be carefully regulated to prevent uncontrolled inflammatory responses and subsequently to promote resolution of inflammation. The suppressor of cytokine signaling (SOCS) proteins play a key role in regulating macrophage phenotype. In this review, we summarize research to date from mouse and human studies on the role of the SOCS proteins in determining the phenotype and function of macrophages. We will also touch on the influence of the SOCS on dendritic cell (DC) and microglial phenotype and function. The molecular mechanisms of SOCS function in macrophages and DCs are discussed, along with how dysregulation of SOCS expression or function can lead to alterations in macrophage/DC/microglial phenotype and function and to disease. Regulation of SOCS expression by microRNA is discussed. Novel therapies and unanswered questions with regard to SOCS regulation of monocyte–macrophage phenotype and function are highlighted.

## Introduction: The Suppressors of Cytokine Signaling as Regulators of Signaling Responses

**Figure 1:**
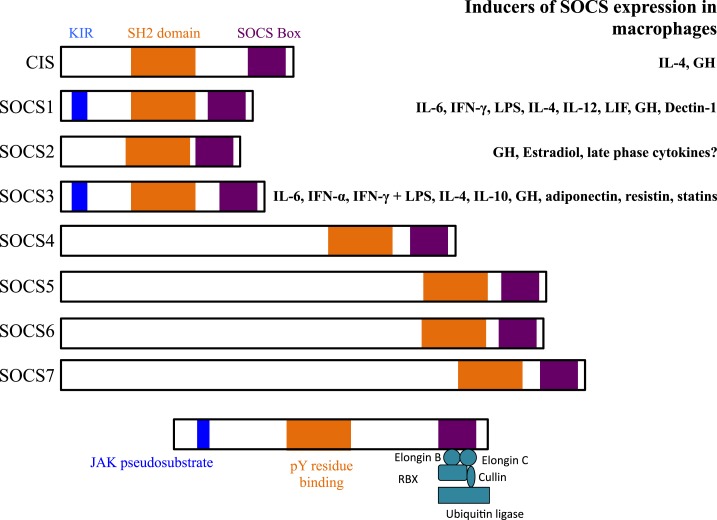
**Structures of suppressor of cytokine signaling (SOCS) family members and known inducers in monocytes/macrophages and DCs**. The SH2 domain is highly conserved across all SOCS members and binds phosphorylated tyrosine (pY) resides on target proteins. The kinase inhibitory region (KIR) of SOCS1 and SOCS3 acts as a pseudosubstrate to block JAK activation. The KIR inhibits the catalytic activity of JAKs by binding to the activation loop of the catalytic domain through both its KIR and SH2 domains. The SOCS box interacts with a complex containing elongin B, elongin C, cullin-5, RING-box-2 (RBX2), and E2 ligase. SOCS box-containing molecules function as E3 ubiquitin ligases and mediate the degradation of proteins that they associate with through their amino-terminal regions. SOCS proteins target the entire cytokine-receptor complex, including Janus kinase (JAK) proteins and SOCS protein themselves, for proteasomal degradation. GH, growth hormone.

The suppressor of cytokine signaling (SOCS) proteins are a family of eight intracellular cytokine-inducible proteins [SOCS1–SOCS7 and cytokine-inducible SH2-containing protein (CIS)] (1, 2). SOCS are expressed basally in every cell and are rapidly induced by a variety of stimuli, including cytokines, toll-like receptor (TLR) ligands, immune complexes, hormones, and in response to glucose (Figure 1) (3). All SOCS family proteins contain an Src homology 2 (SH2) domain that binds phosphorylated tyrosine residues on target proteins, a variable length amino-terminal domain and a conserved carboxy-terminal SOCS box motif that interacts with ubiquitin–ligase machinery (4).

**Figure 2:**
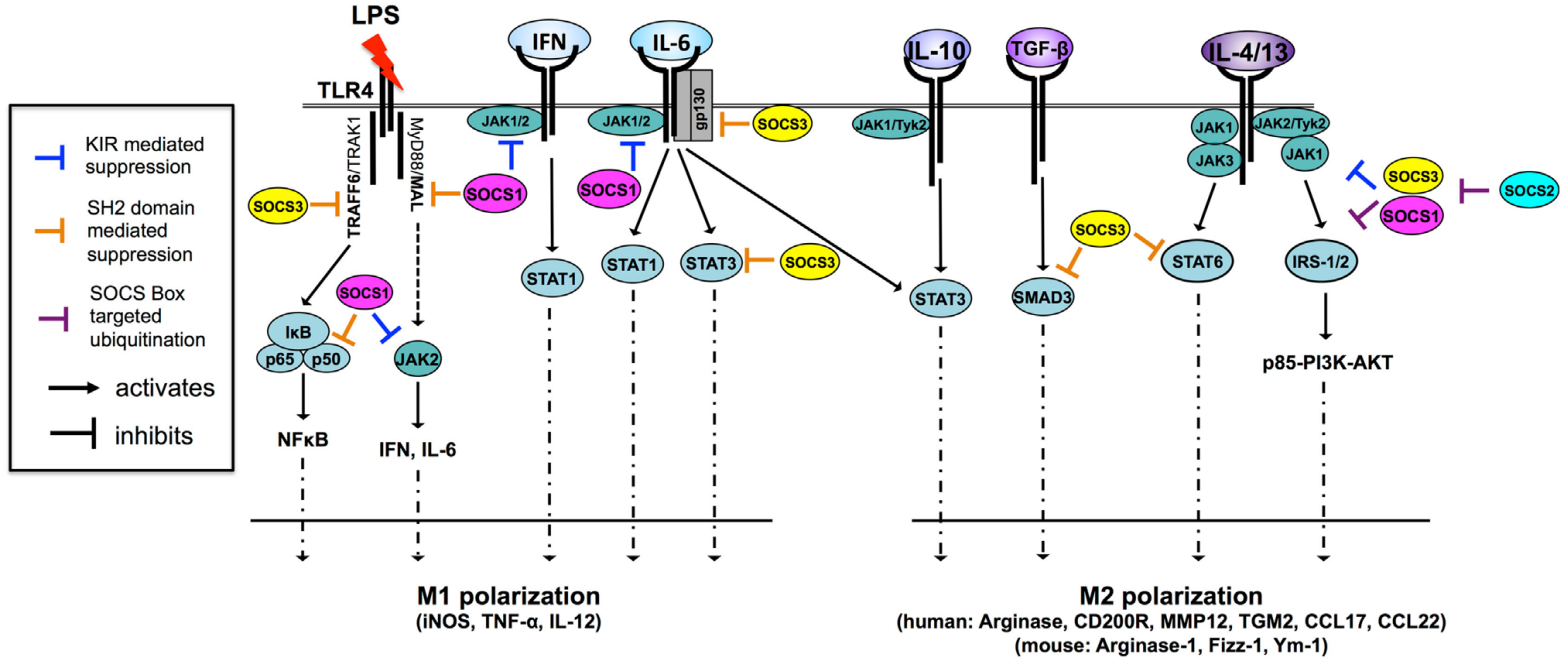
**SOCS1 and SOCS3 are potent regulators of cytokine signaling and macrophage polarization through multiple mechanisms [adapted from Ref. (11)]**. Macrophages are M1 polarized in response to a number of TLR ligands and cytokines. Both SOCS1 and SOCS3 regulate TLR-4 responsiveness through the inhibition of JAK2, MAL, and NF-κB in the case of SOCS1 and through the inhibition of TRAF6 in the case of SOCS3. SOCS1 also regulates IFN- and IL-6-driven M1 polarization by inhibiting JAK activity through the KIR pseudosubstrate domain. SOCS3 regulates IL-6-driven M1 polarization by binding of pY759 the IL-6 receptor gp130 subunit and termination of signaling. SOCS3 also binds activated STAT3 to terminate signaling. Paradoxically, SOCS3 does not inhibit IL-10 signaling because it cannot bind the IL-10 receptor, nor does it effectively bind IL-10-activated STAT3, suggesting SOCS3 binding to STAT substrate is a highly specific and the determinants of this interaction are not fully understood. SOCS3 promotes M1 polarization and regulates TGF-β-driven M2 polarization by binding and preventing nuclear translocation of SMAD3. IL-4 and IL-13 trigger two distinct M2-polarizing pathways, the STAT6 and IRS-2 pathways. SOCS3 regulates IL-4/-13-driven STAT6 activation and nuclear translocation while both SOCS1 and SOCS3 can dampen PI3K and AKT activation by targeting IRS signaling proteins for proteasomal degradation. SOCS2 regulates SOCS1 and SOCS3 expression levels through proteasomal degradation. IRS, insulin receptor substrate; JAK, Janus kinase; MAL, MyD88-adaptor-like protein; STAT, signal transducers and activators of transcription; TGF-β, transforming growth factor-β; TRAF6, TNF-receptor-associated factor 6.

All eight SOCS family members negatively regulate Janus kinase (JAK)/signal transducer and activator of transcription (STAT) signaling through association with key phosphorylated tyrosine residues on JAK proteins and/or cytokine receptors (Figure 2) (3, 5). In addition, SOCS1 and SOCS3 contain a kinase inhibitory region (KIR) that is able to directly suppress JAK tyrosine kinase activity by acting as a pseudosubstrate, binding in or near the activation loop (5–7). SOCS1, SOCS2, and SOCS3 have been shown to negatively regulate signaling through the degradation of signaling molecules via the E3 ubiquitin ligase activity of the SOCS box and ubiquitin–proteasome pathway (8–10). There are many good reviews that discuss the SOCS structure–function relationship in depth (1).

In macrophages, SOCS expression is very low, however, both are rapidly induced upon activation. SOCS1 and SOCS3 have been shown to regulate M1 and M2 macrophage polarization (12–15). M1 and M2 macrophage polarization refers to the phenotype and function of macrophages exposed to either Th1- or Th2-type cytokines. This description was largely established as a result of *in vitro* exposure to different cytokines, bacteria, viruses, or other factors. However, these definitions of polarization state have been revised in recent years with the realization that macrophages *in vivo* can exist along a spectrum between these particular extremes, depending on the external milieu at different stages of disease processes (16). Nonetheless, M1 macrophages can be considered distinct in that they mediate defense against bacterial pathogens, viruses, and tumors, whereas M2 macrophages fight helminth worm parasites and promote wound healing. There are also characteristic transcription factors associated with differentiation into each phenotype. STAT1 and interferon regulatory factor (IRF)5 are associated with M1 macrophages and STAT6 and IRF4 with M2 macrophages. The categorization of M1 and M2 macrophages *in vitro* and *in vivo* has lead to efforts to characterize typical “marker genes,” reviewed extensively elsewhere (17). Herein, we aim to highlight the current understanding of the role of the SOCS in macrophage polarization, with particular emphasis on SOCS1 and SOCS3. We will also touch on SOCS regulation of dendritic cell (DC) polarization and function and their role in polarization of microglia, the resident macrophages of the brain, since these cells are important in neuroinflammation and often overlooked. The role of microRNAs (miRNAs) in regulating SOCS expression and therapeutic potential of the SOCS proteins to direct macrophage and DC function in disease will be discussed.

## SOCS3 Regulates M1 Macrophage Polarization

Researchers have studied how SOCS proteins contribute to the regulation of macrophage polarization *in vitro* by using macrophages from total and conditional knockout (KO) animals and siRNA knockdown approaches.

The loss of SOCS3 in every cell of the body results in death during embryonic development. It causes defects in erythropoiesis in the fetal liver resulting in increased erythrocytosis (18). Additionally, the placenta does not develop properly, owing to lack of regulation of leukemia inhibitory factor signaling in the trophoblast giant cells (19). Fortunately, cell-type-specific KO of SOCS3 in macrophages is not lethal. When SOCS3 was deleted with LysM-cre-driven excision of the *Socs3* allele in mouse macrophages, lipopolysaccharide (LPS) exposure resulted in enhanced M1 polarization, as measured by induction of messenger RNA (mRNA) expression for interleukin (IL)-1β, IL-6, IL-12p40, IL-23p19, and inducible nitric oxide synthase [iNOS (12)]. M1 gene expression was not determined in response to LPS plus interferon (IFN)-γ. Similar findings of enhanced M1 gene expression in response to LPS were observed in alveolar macrophages from LysM-cre-*Socs3*^fl/fl^ mice (20). Interestingly, LPS reduced IL-10 secretion in the *Socs3*-KO macrophages. Both STAT1 and STAT3 activation were increased in response to IFN-γ and LPS. Although the Western blot results appear to show that SOCS1 protein expression increased in the LysM-cre-*Socs3*^fl/fl^ macrophages after either IFN-γ or LPS stimulation, the authors maintain that there was no compensatory increase in SOCS1 expression in these cells. Responsiveness to IL-4, as determined by phosphorylation of STAT6, was not altered, pointing to a minimal role for SOCS3 in regulating IL-4 signaling and M2 polarization. Functionally, the LysM-cre-*Socs3*^fl/fl^ macrophages exhibited higher phagocytic capacity, a typical M1 macrophage function, than did the control *Socs3*^fl/fl^ cells. When the *Socs3*-deficient macrophages were polarized with IFN-γ and LPS and were incubated with naïve T cells, they induced Th1- and Th17-polarization more efficiently than did the wild-type macrophages. The data from this study point to SOCS3 behaving as a negative regulator of the M1 polarization program in mouse macrophages.

Studies in rat bone marrow-derived macrophages (BMM) transfected with siRNA to *Socs3* led to opposite conclusions SOCS3 is required for polarization of macrophages to the M1 phenotype (14). After stimulation with IFN-γ/LPS, expression levels of typical M1 markers, CD86 and IL-6, were reduced and nitric oxide (NO) production was diminished, despite mRNA for *iNOS* being unchanged. IFN-γ + LPS-induced signaling responses, as determined by nuclear STAT3 phosphorylation, were augmented in the SOCS3-knocked down cells, with no impact on the activation of STAT1. This finding contrasts with mouse macrophages from the SOCS3 conditional KO described above (12). Both STAT1 and STAT3 signalings were enhanced and prolonged in the mouse SOCS3^−/−^ macrophages in response to IFN-γ and to LPS, although these stimuli were given separately and not combined in the mouse study. In SOCS3-knocked down rat macrophages, there was an increase in SOCS1 expression that the authors hypothesized was most likely due to increased STAT3 binding to STAT3 sites in the *Socs1* promoter (21). Activated STAT3 in the SOCS3-knocked down cells resulted in increased M2a gene (mannose receptor and arginase) expression in response to IFN-γ/LPS. Furthermore, IL-10 secretion in response to IFN-γ + LPS was maintained in the SOCS3-knocked down rat cells. This observation contrasts with the mouse SOCS3^−/−^ macrophages, which demonstrated reduced IL-10 secretion in response to LPS stimulation alone (12). The responsiveness of M1-polarized (IFN-γ/LPS-treated) rat macrophages lacking SOCS3 to a subsequent IL-4 stimulus was restored. Taken together, this study in rat macrophages indicated that SOCS3 is required for or is a positive regulator of M1 polarization in rat macrophages.

These studies suggest that there may be species-specific differences in how SOCS3 regulates M1 polarization between the mouse and rat macrophages. Alternatively, complete deletion of SOCS3 in the LysM-cre-*Socs3*^fl/fl^ macrophages may result in compensatory alterations in other signaling pathways or proteins that are not observed when the function of SOCS3 is transiently knocked down by siRNA. As the studies described here had opposing results, drawing conclusions about the role of SOCS3 in macrophage polarization will require more investigation with attention to the species from which the macrophages are derived and the stimuli used to elicit M1 polarization.

## SOCS1 Fine-Tunes M1 and M2 Macrophage Differentiation

SOCS1 regulates IFN-γ, LPS and IL-4 signaling and thus participates in regulation of both M1 and M2 macrophage polarization. SOCS1 deficiency in mice leads to death at approximately 3 weeks due to severe inflammation in multiple organs (22, 23), making studying macrophages from these animals challenging. Therefore, to examine the role of SOCS1 in macrophage polarization, macrophages from SOCS1-KO animals crossed with IFN-γ-deficient mice have been used (24).

SOCS1 is necessary to limit the M1 phenotype in response to IFN-γ/LPS stimulation. Macrophages from mice deficient in SOCS1 (and IFN-γ) were hypersensitive to LPS signaling, as shown by enhanced I-κB and p38 phosphorylation (25), demonstrating the role of SOCS1 as a negative regulator of TLR-4 signaling. SOCS1 deficiency in these macrophages resulted in enhanced M1 gene expression (TNF-α, IL-1β, and IL-6) after LPS stimulation. A simultaneously published paper also demonstrated elevated M1 gene expression (TNF-α, IL-12p40) in SOCS1-deficient macrophages stimulated with LPS (26). SOCS1 is also a critical regulator of signaling activated by IFN-β. STAT1 activation in SOCS1-deficient mice was prolonged, and the loss of SOCS1 inhibition of TYK2 was implicated (27).

Consistent with the SOCS1-KO mouse macrophages, SOCS1 knockdown in rat macrophages lead to increased expression of typical M1 genes [major histocompatibility complex (MHC) II, CD86, IL-6, and IL-12p40] in response to IFN-γ/LPS owing to the lack of this important negative regulator (13). Although no signaling intermediates were examined in this study, these results are logical, as SOCS1 is known to inhibit both the MyD88-dependent and -independent pathways downstream of TLR4. SOCS1 has been shown to bind interleukin-1 receptor-associated kinase (IRAK) 1 in an overexpression system, so it is possible that IRAK1 is degraded to suppress LPS-induced signaling (26). SOCS1 directly binds NF-κB p65, leading to its proteolysis and suppression of NF-κB activation (28). The adapter protein, TIRAP/Mal, links receptor engagement of TLR2 and 4 to the MyD88-dependent signaling pathway and is required for full M1 cytokine production in response to LPS (29, 30). Overexpressed SOCS1 interacted with Mal and mediated its degradation (31). Mice deficient in *SOCS1* have unrestrained Mal-induced NF-κB signaling and proinflammatory cytokine production after LPS stimulation (31). Although the absence of SOCS1 led to increased expression of many typical M1 genes, expression of iNOS was diminished after IFN-γ/LPS stimulation of SOCS1 siRNA-transfected rat macrophages (13). Interestingly, expression of typical M2 genes (arginase I and IL-10) was enhanced in the *Socs1*-knockdown cells in response to IFN-γ/LPS stimulation.

SOCS1 plays a negative regulatory role in IL-4 signaling and M2 macrophage polarization. This was demonstrated by studies in BMM from *Socs1*-KO mice that expressed more arginase I after IL-4 stimulation (32). However, using rat BMM transfected with siRNA to *Socs1*, Whyte et al. (13) demonstrated that exposure to IL-4 caused decreased expression of arginase I, a hallmark M2 gene, and increased expression of iNOS, characteristic of M1 macrophages. In the rat SOCS1-knocked down cells, three other characteristic M2 macrophage genes that they tested, macrophage mannose receptor (*Cd206*), *Chi3l3* (YM1), and *Retnla* (FIZZ1), were unaffected by SOCS1 knockdown. To attempt to determine the molecular mechanism, the authors described SOCS1 knockdown resulting in diminished phosphorylation of serine 473 of AKT after 30 and 60 min of IL-4 stimulation. Phosphorylation of AKT at serine 473 was used as a surrogate measure for PI3K activity. SOCS3 expression increased in the SOCS1-knocked down cells, suggesting that counter-regulation of SOCS1 and SOCS3 expression dictates macrophage polarization. Inhibition of PI3K activity (decreased phosphorylation of AKT Ser473) was attributed to this increase in SOCS3 expression. However, none of the signaling analysis presented was reported as statistically significant, and the analyses were carried out only to 60 min; thus any effect on the downregulation of signaling in the SOCS1-knocked down cells may have been missed. Similar to the findings from *Socs3* siRNA knockdown in rat macrophages, the SOCS1-knocked down rat macrophages lost IL-4-mediated attenuation of subsequent IFN-γ/LPS responses. These data show that IL-4-induced SOCS1 participates in the refractory state of macrophages to IFN-γ/LPS after the cells have been previously exposed to IL-4.

Taken together, the data described above suggest that, unlike SOCS3, SOCS1 regulates both M1 and M2 polarization in rat macrophages *in vitro*. Furthermore, there appear to be some species-specific differences in how SOCS1 and SOCS3 control macrophage differentiation in response to IL-4 and IFN-γ/LPS, as the rat and mouse macrophage studies drew opposite conclusions. This species-specific difference is presented in Table 1.

**Table 1:** **Summary of SOCS1 and SOCS3 KO, conditional KO, and siRNA knockdown studies to determine their role in polarization of mouse, rat, and human macrophages**.

	Mouse BMM				Rat BMM				Human macrophages			
	Stimulus	Readout	Conclusion	Reference	Stimulus	Readout	Conclusion	Reference	Stimulus	Readout	Conclusion	Reference
**SOCS1**												
Total KO^a^	LPS	↑TNF-α, NO, IL-6/IL-1β, pI-κB, p-p38, pJNK	Negative regulator of M1 (TLR signaling)	Kinjyo et al. (25)								
	IL-4	↑ArgI	Negative regulator of M2 (IL-4 signaling)	Dickensheets et al. (32)								
	IFN-α	↑pSTAT1	Negative regulator	Fenner et al. (33)								
	IFN-β	↑pSTAT1	Negative regulator	Gingras et al. (27)								
Conditional KO	LPS, palmitate	↑M1 genes	Negative regulator of M1 (TLR signaling)	Sachithanandan et al. (34)								
siRNA knockdown					IFN-γ + LPS	↑M1 genes, exc. iNOS	Negative regulator of M1	Whyte et al. (13)	Not determined			
					IL-4	↓ArgI, but not other M2 genes	Positive regulator of M2					
**SOCS3**												
Total KO	Not determined											
Conditional KO	IL-6 + LPS	↑pSTAT3 ↓TNF-α, IL-12	Negative regulator of IL-6 signaling	Yasukawa et al. (35)^b^								
	IL-10 either ± LPS	No Δ in pSTAT3 or TNF-α, IL-12 production	Not a negative regulator of IL-10 signaling									
	IL-6	↑pSTAT1/3 ↑M1 genes	Negative regulator of M1	Qin et al. (12)								
	LPS	↑pSTAT1/3	Negative regulator of M1									
		↑M1 genes										
		↑Phagocytosis										
	IFN-γ	↑pSTAT1/3		Qin et al. (12)								
	IL-4	No Δ in pSTAT6	Not a negative regulator of IL-4 signaling/M2									
	LPS	↑M1 genes	Negative regulator of M1	Yan et al. (20)^c^								
siRNA knockdown					IFN-γ + LPS	↓M1 genes, exc. iNOS	Positive regulator of M1	Liu et al. (14)	IFN-γ + LPS	↓M1 genes	Positive regulator of M1	Arnold et al. (15)
						↑pSTAT3						
						↑SOCS1						

*The experimental approach is listed in the left-hand column. ^a^The SOCS1 knockout studies utilized the SOCS1^−/−^ IFN-γ^−/−^ animals due to the early mortality of the SOCS1^−/−^ animals. The majority of these studies used bone marrow-derived macrophages, except when ^b^thioglycollate-elicited and ^c^alveolar macrophages were employed. There were no studies in macrophages from SOCS3^−/−^ mice, as this deletion is embryonic lethal. The conditional knockout studies all used macrophages from LysM-cre animals crossed to the SOCS1^fl/fl^ or SOCS3^fl/fl^ animals. p = phospho*.

## Regulation of Human Macrophage Polarization by the SOCS Proteins

Regulation of human macrophage polarization by the SOCS proteins has not been well studied. Dickensheets et al. (32) described induction of mRNA for *SOCS1* in response to IL-4, IL-13, IFN-β, and IFN-γ in human peripheral blood monocytes. SOCS3 knockdown with siRNA in human macrophages resulted in decreased expression of M1 cytokines (IL-1β, IL-6, IL-12p70, and IL-23) in response to IFN-γ/LPS stimulation (15). This observation is consistent with the SOCS3 knockdown effect on M1 genes in rat macrophages. Similarly, SOCS1 expression and the amount of activated STAT3 increased in the LPS-stimulated SOCS3-knocked down human macrophages. The consequence of knocking down SOCS1 on the polarization of human macrophages is unknown. Furthermore, the majority of studies examining SOCS1 and SOCS3 rely on expression of mRNA for *SOCS1* and *SOCS3* to draw conclusions about SOCS protein expression and function. The expression of the SOCS proteins, however, is tightly regulated by translation mechanisms (36), as well as post-translational modifications (37). The amount of protein expression is rarely validated in many studies. Future endeavors should focus on a more careful characterization of SOCS *protein* expression and how these proteins regulate human macrophage polarization in health and disease.

## Impact of SOCS Deficiency in Macrophages in Mice

In terms of the functional effects of SOCS1 loss *in vivo*, mice deficient in SOCS1 (and IFN-γ) have heightened sensitivity to LPS-induced shock and increased production of inflammatory cytokines (25, 26). These observations are consistent with the role of SOCS1 in restraining proinflammatory signaling. Similar results validate SOCS1 as a negative regulator of proinflammatory signaling in the methylated bovine serine albumin (mBSA)/IL-1-dependent model of arthritis in SOCS1^−/−^ IFN-γ^−/−^ mice (38). The data from SOCS1-deficient mice in the ovalbumin model of asthma, a Th2-/M2-type disease, support the role of SOCS1 as a negative regulator of M2 polarization (39). Serum IgE, eosinophilia, Th2-cytokine expression, and arginase I were increased in the lungs of the KO mice compared to the control animals.

Mice with a macrophage-specific deletion of SOCS1 displayed hypersensitivity to administered LPS and palmitic acid with an elevation of proinflammatory cytokines and systemic inflammation (34). Macrophage accumulation in the liver and liver inflammation resulted in hepatic insulin resistance in these animals, highlighting the role of SOCS1 in restraining M1 inflammatory responses, while promoting M2 macrophage polarization. Mice with a SOCS1 deficiency in their macrophages had a lower bacterial load in the lung a week after infection with *Mycobacterium tuberculosis* than did mice with normal SOCS1 expression. This effect was attributed to enhanced IFN-γ production and expression of iNOS (expression of mRNA was measured) and suggests that SOCS1 restrains M1 polarization in macrophages (40). The LysM-cre-*Socs1*^fl/fl^ mice also exhibited less infection-induced lung inflammation than did the SOCS1-KO animals after the initial acute phase of infection. This observation led the authors to conclude that non-macrophage SOCS1 expression was critical to control inflammation resulting from infection 3 weeks later. Consistent with an enhanced M1 phenotype, deficiency of SOCS1 in macrophages rendered LysM-cre-*Socs1*^fl/fl^ mice resistant to B16 melanoma cell implantation and protection from dextran sulfate sodium (DSS)-induced tumor formation in the colon (41).

Studies to define SOCS3 as a positive or negative regulator of M1 polarization using mice with a macrophage-specific deletion of SOCS3 have yielded differing results *in vivo*. For example, when the LysM-cre-*Socs3*^fl/fl^ animals were challenged with LPS, two different outcomes were observed: protection from or exacerbation of LPS-induced septic shock (12, 35, 42). The different outcomes may have been due to differences in the concentration of LPS used for challenge. Another example of unexpected *in vivo* phenotypes in mice with a deletion of SOCS3 in macrophages was the increased mortality and pathogen burden after *Toxoplasma gondii* infection in LysM-cre-*Socs3*^fl/fl^ mice (43). The authors found that SOCS3-deleted macrophages demonstrated uncontrolled IL-6 signaling that suppressed IL-12 production, a protective cytokine against toxoplasmosis. From the *in vitro* studies in SOCS3-deficient macrophages described previously, the opposite outcome would have been predicted – that deletion of SOCS3 would increase M1 gene expression and protect from the infection. However, the complexity of the inflammatory response *in vivo* shows that predictions based on macrophage behavior *in vitro* do not necessarily perfectly translate to whole animal models of disease. The caveat is that each disease model must be dissected individually to determine the critical cytokines and the relative importance of the macrophage to pathology.

Other mouse models of disease support SOCS3 as a negative regulator of M1 responses. For example, in the mBSA model of rheumatoid arthritis (RA), proinflammatory responses were elevated in the absence of SOCS3 in the hematopoietic and endothelial cell compartment (44). BMM from these SOCS3^−/*Δvav*^ mice could not regulate IL-6 production induced by IL-1. LysM-cre-*Socs3*^fl/fl^ mice were also vulnerable to neuroinflammation characterized by infiltration of immune cells into the central nervous system (CNS) in a myelin oligodendrocyte glycoprotein-induced experimental autoimmune encephalomyelitis (EAE) model (45). Another example of enhanced M1 responses in the absence of SOCS3 in macrophages *in vivo* is protection from tumor transplantation and metastasis (46), similar to macrophage-specific SOCS1 deficiency. Tumor protective responses in macrophages are usually associated with an M1 macrophage phenotype. Surprisingly, a deeper analysis of the tumor-associated macrophages (TAMs) isolated from the B16 tumors in these animals revealed a mixed M1/M2 phenotype. Expression of M1 markers (TNF-α, IL-6, IL-12p35), with the exception of iNOS, was decreased in the SOCS3-deficient cells, relative to that of wild-type macrophages. Despite upregulation of typical M2 markers (arginase I and IL-10), usually associated with tumor progression, SOCS3-deficient macrophages provided tumor protection because of enhanced MCP-2/CCL8 production. The mechanism by which MCP-2 brought about tumor protection was not determined in any molecular or cellular detail however. The results of this *in vivo* study underscore that ascribing M1 macrophages as tumoricidal may be over simplistic, as the SOCS3-deficient macrophages had lower expression of M1 markers and increased M2 markers yet afforded tumor protection. Furthermore, the existence of a mixed M1 and M2 phenotype in macrophages *in vivo* highlights the difficulty of assigning “typical markers” and unequivocal phenotyping *in vivo*. An additional complexity arises from changing macrophage phenotypes over the course of the disease *in vivo*. Depending on when the pathological tissue is sampled, the macrophages will have different phenotypes at initiation, during, and in the resolution phase of the disease.

## The Role of Macrophage SOCS Expression in Human Disease

Because the balance between SOCS1 and SOCS3 expression can regulate the polarization of macrophages, enhanced or suppressed expression of these two regulators under conditions of infection or disease could affect the differentiation of macrophages and either contribute to or diminish disease pathology. An altered balance of SOCS1/3 expression in monocytes and/or macrophages has been described in several human infections and disease states. For example, expression of *SOCS1* mRNA and protein was higher in monocyte–macrophages isolated from the blood of patients with chronic hepatitis C infection than in those from healthy controls in response to LPS/R848 stimulation (47). In this setting, hepatitis C virus core protein induced SOCS1 expression, which in turn induced PD-1 to suppress IL-12 production by primary human monocyte–macrophages. Knocking down SOCS1 in the human monocytic cell line THP-1 diminished PD-1 expression and restored IL-12 secretion. Bacterial infections also subvert SOCS expression in macrophages to prevent efficient M1 polarization and clearance by the host immune system. Patients with active and severe pulmonary tuberculosis displayed higher IL-4 and CCL4 expression in the bronchoalveolar lavage (BAL) fluid and greater SOCS3 expression in cells from BAL than did patients with mild forms of tuberculosis (48).

In situations of disease, altered expression of SOCS mRNA and protein in macrophages may be a response to a particular inflammatory environment, rather than a distinct dysregulation of SOCS expression in macrophages. For example, the macrophages in synovial fluid of inflamed knee joints from patients with RA expressed more mRNA for *SOCS1*, *SOCS3*, and *CIS* than did peripheral blood monocytes from the same patients or healthy controls (49). This comparison led the authors to conclude that it was the inflammatory environment of the RA joint to which the macrophages were responding that caused changes in SOCS expression. Similarly, White et al. (50) undertook an immunohistochemical analysis of SOCS3 expression in CD68+ macrophages/DCs in tissue from patients with seven inflammatory conditions. They found a statistically significant increase in SOCS3 expression in the CD68+ cells from patients with lymph node sarcoidosis, Crohn’s disease, ulcerative colitis [also noted by Miyanaka et al. (51)], acute appendicitis, and temporal arteritis, compared to that in adjacent normal tissue. Polymorphisms in the SOCS genes that correlate with disease or disease severity have been described in a number of different diseases (52–56). Because SOCS expression affects macrophage polarization and function so critically, it can be appreciated that polymorphisms that affect SOCS expression or function might positively or negatively influence diseases in which polarized macrophages play either a pathogenic or protective role. For example, the SOCS1 promoter SNP, SOCS1-820T SNP, decreased the ability of yin yang-1 (YY1) to suppress transcription of SOCS1 (53). Whether SOCS genetic polymorphisms such as this one affect the polarization or function of macrophages in particular has not been explored. Similar to the *in vitro* work on human macrophages, future studies on the role of the SOCS in human disease should focus on determination of differential expression of SOCS *protein*, rather than mRNA. The temporal changes in macrophage phenotype and SOCS protein expression during the course of disease are important and must be evaluated before drawing conclusions about the function of these regulators in human pathological disease processes.

## SOCS, Dendritic Cells, and T Cell Priming

Dendritic cells play an important role in the initiation and coordination of immune responses through antigen presentation and interaction with effector innate and adaptive lymphocytes. DCs are highly motile and uniquely able to prime naïve T cells. Inappropriate DC function has been implicated in autoimmune diseases such as systemic lupus erythematosus (SLE) (57), multiple sclerosis (MS) (58), type 1 diabetes (T1D) (58), and allergy (59). Upon activation, DCs upregulate co-stimulatory molecules CD80, CD86, CD40, MHC I, and MHC II for antigen presentation, inflammatory cytokines, and chemokine receptors. In addition, like macrophages, DCs upregulate SOCS in response to stimuli (60). *Ex vivo*, DCs deficient in SOCS1 are more sensitive to TLR ligand stimulation, secrete more proinflammatory cytokines, have enhanced antigen presentation capacity, and induce greater T cell proliferation than DCs expressing functional SOCS1 (34, 61–64). *In vivo*, selective deletion of SOCS1 from DCs results in priming of lethal autoimmune CD8 T cell responses (65). The enhanced CD8 T cell priming was not attributed to increased antigen presentation but rather increased and prolonged IL-12p35 production by DCs. This process was at least partially attributed to autocrine IL-12 signaling in DCs and was reversed in IL-12-deficient DCs. The increased IL-12 production similarly enhanced Th1-polarized, but not Th17-polarized CD4 T cell responses and inhibited Th2 development. These findings, taken together with *in vivo* observations in KO mice, indicate that in DCs, SOCS1 limits T cell activation and the development of lethal T cell-driven autoimmune diseases by regulating the amount and duration of type 1 T cell polarizing cytokine signaling. In humans, decreased SOCS expression in circulating PBMCs correlates well with autoimmune disease severity. Polymorphisms in SOCS1 that cause non-functioning or low functioning SOCS1 are a risk factor for MS (56, 66, 67), SLE (68, 69), and asthma (54).

The role of SOCS3 in DCs remains less well defined. Certainly SOCS3 is a critical negative regulator of IL-6 and IFN-γ signaling; however, its effect on co-stimulatory molecule expression, antigen presentation, and T cell priming is minimal. Instead, SOCS3 expression by DCs plays a critical role in controlling the balance between T cell activation and tolerance through the regulation of indoleamine 2,3-dioxygenase (IDO) (70, 71). Orabona et al. investigated the role of SOCS3 in regulating IDO in DCs. They reported that in response to immunogenic DC stimulation, SOCS3 has the ability to bind IDO and is responsible for its ubiquitin-mediated proteasomal degradation (70). These findings implicate a dual role of SOCS3 in regulating T cell responses through negative regulation of IDO in DCs. In humans, SOCS3 expression is lower in circulating monocytes from patients with active-relapsing MS than in monocytes from healthy individuals or those with MS in remission (72), supporting the role for SOCS3 in immune suppression. Because SOCS3 does not appear to regulate T cell priming, the observation that SOCS3 regulates IDO expression may provide clues as to the role of SOCS3 in maintaining immune suppression during ongoing autoinflammatory disorders.

## SOCS2 and CIS in Macrophage and Dendritic Cell Polarization and Function

Less is known about the function of SOCS2 and CIS. Both SOCS2 and CIS are closely related, and lack the N-terminal KIR domain, meaning the inhibitory function is dependent on competitive binding via its SH2 domain and the ubiquitination/proteasomal degradation. Both SOCS2 and CIS are expressed in monocytes/macrophages as well as DCs; however, there is a distinct lack of mechanistic studies available to adequately describe their relative importance in controlling macrophage polarization. Like SOCS1 and SOCS3, SOCS2 has E3 ubiquitin ligase activity (73) and uniquely has the ability to target SOCS1 and SOCS3 (74, 75), as well as growth hormone (GH) receptor (76–78) for degradation. Furthermore, SOCS2 regulates epidermal growth factor and insulin like growth factor (IGF)-1 signaling through the regulation of STAT5B. It was recently identified that SOCS2 expression is induced by estradiol to limit GH-driven JAK1 phosphorylation (79). In line with these findings, SOCS2 mRNA expression reportedly increased immediately prior to ovulation in bovine, suggesting that SOCS2 can be induced by estrogen. It is important to note that the increase in SOCS2 mRNA also mirrored IGF-1 levels. Since SOCS2 is known to regulate IGF-1, this three-way interaction may merit further investigation to better understand the effects of sex hormones on the regulation of the SOCS family members. This may have important implications in macrophage biology as SOCS2 may regulate macrophage polarization through the IGF-1 signaling axis although this remains to be investigated. In LPS-matured human DCs, SOCS2 induction is delayed compared to SOCS1/SOCS3 (80, 81) and is actually indirectly induced by TLR-4 stimulation through the induction of type I IFNs (82). Transient siRNA knockdown of SOCS2 dampened TLR-4 driven MAPK signaling, proinflammatory cytokine release and DC maturation, suggesting that SOCS2 may actually be a positive regulator of monocyte maturation (80). Alternatively, knockdown of SOCS2 allowed SOCS1 and/or SOCS3 to accumulate leading to increased regulation of TLR signaling. By contrast, murine DC maturation was not affected by loss of SOCS2 (83). However, whole body SOCS2^−/−^ mice are more susceptible to inflammation-induced lethal pathology triggered by some [*T. gondii* (84)] but not all [*Mycobacterium bovis* (85)] macrophage-targeting intracellular infections. These discrepancies may reflect the ability of the pathogen to trigger TLR-ligand-driven type I IFN responses (82) as well as the availability of SOCS1/SOCS3 to balance inflammatory responses. Taken together, it is clear that SOCS2 acts as a growth regulator both by directly inhibiting signaling and by inhibiting the negative regulators, SOCS1 and SOCS3. However, the role of SOCS2 in regulating macrophage/DC inflammation appears to be species specific, with human cells showing altered maturation and effector function (86, 87) while murine cells are not always affected. The biological role of SOCS2 is subtle, with KO mice developing mild abnormalities and very few human polymorphisms described to date. To date, much of our understanding of SOCS2 comes from *in vitro* analyses and the reported effects of SOCS2 are dependent on the specific cell/organ being examined as well as the dose. This would suggest that either SOCS2 is partially redundant or SOCS2 regulation of SOCS1/3 plays a bigger role than currently appreciated.

Cytokine-inducible SH2-containing protein (CIS) is the oldest of the SOCS family members. Like SOCS2, CIS is induced by GH, IGF-1, growth factors M-CSF, GM-CSF, and by multiple cytokines (88–90). In DCs, CIS regulates growth and proliferation through the inhibition of STAT5 (88, 90) in order to allow for complete differentiation. Knockdown of CIS impaired antigen presentation and OTI/OTII proliferation through the downregulation of MHC and co-stimulatory molecules as well as reduced Th1 polarizing cytokines IL-12, TNF-α, and IL-6 (91). Interestingly, knockdown of CIS also had no appreciable effect on Th2 polarization (91). In line with these findings, knockdown in CIS impaired vaccine-induced responses, CTL development, and anti-tumor immunity (91). Human polymorphisms in the *CIS* promoter have been linked with increased susceptibility to (92) pediatric *M. tuberculosis* infection (93). Given the critical role of CIS in antigen presentation, cytokine production, and DC-mediated T cell priming, it is easy to appreciate how decreased CIS expression could interfere with vaccination efforts as well as predispose children to unchecked bacterial dissemination once contracting tuberculosis. The mechanistic role of CIS in promoting type 1-polarized DC/macrophages has not been investigated. In T cells, CIS promotes CTL/Th1 development (94) through unknown mechanisms although it has been proposed that decreased CIS expression may lead to enhanced STAT5 activation, a transcription factor for Tregs, upsetting the Th1/Treg balance. If validated, this could account for at least some of the observed *in vivo* effects. However, *in vitro* knockdown of CIS in DCs clearly support a role for CIS in promoting maturation and cytokine signaling. Given its similarity with SOCS2, it is possible CIS is acting as a negative regulator of other negative regulators, namely SOCS1 and SOCS3. Although there is not currently any evidence to support this theory, SOCS2^−/−^ monocytes/DCs closely resemble CIS-knockdown monocytes/DC. Intense investigation into the immune potentiating mechanisms of SOCS2 and CIS may reveal important clues about immune regulatory mechanisms and provide useful new approaches for immune-driven diseases from autoimmune diseases to cancer immunotherapies.

## SOCS4, SOCS5, SOCS6, and SOCS7 in Macrophage and Dendritic Cell Polarization and Function

As with the previously described SOCS family members, SOCS4, SOCS5, SOCS6, and SOCS7 are expressed by myeloid cells and regulate a variety of cytokine and hormone signaling pathways. Until recently, no direct function could be ascribed to SOCS4 and no animal models were available to being to investigate its role in inflammation. Kedzierski et al. (95) generated mice deficient in SOCS4 and reported normal thymic development and immune system development. However, upon influenza virus infection, these mice rapidly succumbed to cytokine-driven immune pathology. In spite of enhanced chemokine and cytokine responses in the lung and spleen of SOCS4^−/−^ mice, the authors observed decreased CD8 T cell recruitment and impaired viral clearance. *In vitro* analysis of T cell revealed SOCS4^−/−^ CD8 T cells did not respond to polyclonal CD3/CD28 activation, indicating SOCS4 positively regulates T cell receptor signaling. Although preliminary, these findings would suggest that SOCS4 may negatively regulate cytokine/chemokine responses in myeloid and structural cells while the mechanisms of SOCS4 positive regulation of T cell receptor signaling remain to be investigated. Given these observations, detailed analysis of SOCS4 binding partners in macrophages and interactions with other SOCS family members merit consideration.

SOCS5 has been shown to be expressed in CD4 T cells and inhibit Th2 differentiation by binding the IL-4Rα and blocking STAT6 phosphorylation, thus favoring Th1-polarization (96). Global overexpression of SOCS5 lead to enhanced local inflammation in a murine model of bacterial peritonitis (97). Closer examination of infiltrating cells revealed M1-polarized macrophages displaying enhanced phagocytic capability and decreased STAT6 phosphorylation. Taken together, these findings suggest that SOCS5 may promote M1 polarization through the inhibition of IL-4 signaling although this remains to be validated. Furthermore, SOCS5 induction has not been reported in monocytes/macrophages in response to M1 or M2 polarizing conditions, suggesting that SOCS5 plays a minor role in macrophage polarization or SOCS5 functions basally to limit M2 polarization. Systematic evaluation of SOCS5 protein induction may be necessary to determine that SOCS5 plays a meaningful role in macrophage biology.

SOCS6 and SOCS7 negatively regulate insulin signaling through the interaction with insulin receptor substrate (IRS) signaling molecules IRS-4 in the case of SOCS6 (98, 99) and IRS-1 in the case of SOCS7 (100). Given the importance of insulin availability and metabolism in macrophage polarization (101–103) as well as the importance of IRS-1/-2 in IL-4 signaling (104, 105), SOCS6 and SOCS7 may play a yet undefined role in macrophage polarization. More in-depth analysis of the role of these lesser-studied SOCS family members is required.

## SOCS Proteins Regulate Neuroinflammation

Suppressor of cytokine signaling family members have been implicated in regulation of CNS inflammation (106). SOCS1 and SOCS3 have been shown to control inflammatory cytokine signaling in neurons (107–113), Schwann cells (107, 114), oligodendrocytes (115–117), astrocytes (118–120), and microglia (121–128). Microglia are free-moving macrophage-like cells that are the primary immune component of the CNS immune system and carry out immune surveillance, scavenging, phagocytosis, and antigen presentation (129–131). Like M1 macrophages, microglia respond to conditions of infection, trauma, and neuronal death by transiently activation to release TNF-α, NO, and reactive oxygen species to eliminate pathogenic organisms and remove dead cells in the damaged area (132). Prolonged or chronic M1-polarized microglial activation causes loosening of the blood–brain barrier, leukocyte, and lymphocyte influx (132, 133) and it is a hallmark of neurodegenerative diseases, such as amyotrophic lateral sclerosis (ALS), Alzheimer’s, Parkinson’s, and Huntington’s diseases (129, 134, 135). Microglia rapidly return to a resting or M2-like state in order to regulate inflammation, repair, and maintain homeostasis (129, 130, 136, 137). M2-polarized microglia express typical M2 macrophage markers (138, 139), secrete anti-inflammatory factors (140–143), IL-10, TGF-β, neuronal growth factors (144–149), and express SOCS proteins (150), necessary for wound healing. This shift is associated with re-myelination in a rodent model of de-myelinating disease (138), better control of inflammation in a rodent model of ALS (151) and ischemic stroke (152). Failure to shift to a M2 microglia phenotype or a predominance of M1-polarized microglia is associated with worsening tissue damage and neuronal loss (153), suggesting that M2-polarized microglia act as gate-keepers against neuroinflammation.

Apoptotic cells, cytokines, and neurotoxic mediators, such as amyloid beta peptide (154), among others, are known to induce expression of SOCS family members. As a result, investigation has gone into deciphering the role of SOCS in modulating neuroinflammation. The observations discussed below regarding the role of SOCS proteins in neuroinflammation have been made from human disease or from studies carried out in SOCS competent animals rather than SOCS-deficient animals, therefore, the precise inflammatory triggers and downstream signaling pathways have not been delineated in microglia. However, given our extensive understanding of TLR-ligand and cytokine-driven signaling in macrophages, we can certainly extrapolate our understanding of SOCS proteins in regulating macrophage inflammation into neuroinflammation. SOCS1-expressing microglia have been correlated with reduced NO production, decreased sensitivity to TLR ligand stimulation and cytokine-induced signaling, all leading to controlled neuroinflammation (127, 155, 156). Attenuating SOCS1 expression in microglia promoted proinflammatory M1-like microglia and worsening neuroinflammation (157). Taken together, these findings indicate that SOCS1 induction and subsequent M2 polarization are critical for the neuroprotective functions of microglia. Given our understanding of SOCS1 in regulating M1 macrophage polarization, it is likely SOCS1 is performing a similar role in microglia although this remains to be explicitly demonstrated. Interestingly, blood-derived monocytes recruited into the brain also display an M2-polarized phenotype and express high levels of SOCS1. Enhancing SOCS1 expression in the brain is viable therapeutic option for treating acute brain injuries such as stroke and trauma.

Recently, two several reports showing that resveratrol has neuroprotective effects in a mouse model of Parkinson’s disease (158, 159) through the induction of SOCS1 expression in microglia *in vitro* (159). These studies provide evidence that enhancing SOCS1 expression in microglial cells can have a dramatic neuroprotective effects on CNS inflammation and support continued investigation into therapies that increase SOCS1 expression in microglia to control progressive neuroinflammatory diseases.

To date, SOCS1 and SOCS3 appear to be similarly induced and have overlapping roles in microglia, although the kinetics, factors that regulate their expression and discrete roles of SOCS expression in microglia during the course of disease are only partially elucidated. SOCS3 expression has been suggested to be detrimental for axonal growth by inhibiting the production of growth factors (160). Examination of the expression patterns of SOCS after peripheral nerve injury revealed that SOCS1 expression is restricted primarily to macrophages whereas SOCS3 expression is restricted to Schwann cells (114). In this model, SOCS1 expression inversely correlated with phosphorylation of JAK2 and STAT3 and the expression of proinflammatory cytokines IL-1β and TNF-α, whereas SOCS3 expression negatively correlated with the expression of IL-6 and LIF (161, 162). These findings indicate that these molecules regulate discrete aspects of neuronal inflammation and may be restricted to different cell types. Detailed analysis of SOCS1 and SOCS3, as well as other SOCS family members, including SOCS2 and CIS in microglia, will help to elucidate how and when microglia transition from a proinflammatory to immunoregulatory phenotype and how to best exploit this process to protect against neuronal loss and slow chronic degenerative diseases. Moreover, identifying drugs/biologics agents that modulate SOCS expression in the brain may help to combat progressive neurodegenerative as well as acute brain injuries.

## Regulation of SOCS Activity in Macrophages and DCs for Therapeutic Benefit in Disease

Because altering the expression of SOCS1 and SOCS3 can have profound effects on the polarization and function of both macrophages and DCs, therapeutic strategies aimed at augmenting or suppressing expression or function of the SOCS proteins could significantly affect disease progression and pathogenesis. In this regard, researchers have used different approaches to modulate SOCS expression or function *in vitro* and *in vivo* to try to inhibit inflammatory signaling that leads to pathogenesis.

## microRNAs Regulate SOCS Expression

Several mechanisms that regulate SOCS expression have been characterized, including promoter methylation and microRNAs (miRNAs). MiRNAs are small 22–26-nucleotide non-coding RNAs that target mRNA to fine-tune gene expression. Long primary transcripts (primary microRNA) are transcribed by RNA polymerase II, processed by the nuclear enzyme Drosha and released as a hairpin precursor. Precursor microRNA are processed by the RNase III enzyme Dicer to ~22 nucleotides (mature microRNA) and then incorporated into RNA-induced silencing complex (RISC). The microRNA–RISC complex binds the 3′-untranslated region of target messenger RNA (mRNA) and either promotes translational repression or mRNA degradation.

Many miRNAs have been described to regulate SOCS1 and SOCS3 expression. Perhaps the best characterized is mir-155. Mir-155 regulates SOCS1 expression by inhibiting translation and attenuating protein expression (163–166). Consequently, DCs deficient in mir-155 have greater SOCS1 expression, display decreased co-stimulatory molecule expression, have decreased antigen presentation, and have a reduced ability to cause T cell proliferation *in vitro* (167, 168). Animals with mir-155-deficient DCs are less susceptible to the development of autoimmune disease and staphylococcal enterotoxin B-induced acute lung injury (169) and are resistant to allergic airway disease (170). Furthermore, mir-155-deficient apoE^−/−^ mice are partially protected from atherogenic inflammation (171). Overexpression of mir-155 or lentiviral delivery of pre-mir-155 in macrophages partially recapitulates the SOCS1^−/−^ phenotype of increased sensitivity to TLR stimulation and cytokine signaling. It is important to note that mir-155 targets other proteins and that some of its reported biological roles likely occur through a SOCS1-independent mechanism. Taken together, there is compelling evidence that mir-155 is important for fine-tuning SOCS expression and, therefore, has a profound impact on macrophage polarization and function. Other miRNAs are predicted to bind SOCS1, including mir-150 (172), mir-221 (173), mir-572 (174), and mir-19a (175); upregulation of these miRNAs correlates with increased inflammation. In contrast to miRNAs that suppress SOCS1 expression, mir-29b reportedly induces SOCS1 by demethylating its promoter (176). Although these SOCS-regulating miRNAs have been identified in clinical disease, their cell specificity and functional role in macrophages remains uninvestigated.

The miRNAs that regulate SOCS3 expression are less well defined than miRNA regulators of SOCS1. Several miRNAs, mir-19b (177), mir-203 (178), and let-7 (179), are predicted to bind SOCS3 transcripts. Mir-19a, which is also predicted to inhibit SOCS1, has been shown to decrease SOCS3 expression and subsequent IFN-α and IL-6 signaling by regulating the JAK-STAT pathway (180). Balasubramanyam et al. reported that mir-146a expression was decreased in PBMCs from patients with type 2 diabetes. Decreased mir-146a was associated with increased type I inflammation, TNF-α and IL-6 and a trend toward increased SOCS3 expression (181). These changes further correlated with insulin resistance and poor glycemic control. These findings support the current paradigm in which SOCS3 is required for M1polarization, and increased SOCS3 expression may facilitate increased M1-driven inflammation. Since many diseases are drawn-out processes that occur over many years and involve several cell types, the changes in miRNA-driven SOCS expression or repression are likely complex. They cannot be distilled down to a single failed regulatory pathway and their regulatory mechanisms may differ between effector cell types. These findings highlight the distinct role for SOCS family members in regulating discrete pathogenic mechanisms in autoimmune and chronic diseases. Further elucidation is still needed into the differences in mechanisms that regulate miRNA expression in peripheral immune cells and local inflammatory immune cells.

The human genome contains 11 let-7 miRNA genes that produce eight different mature let-7 miRNAs. There are several reports of changes in let-7 miRNA expression in human diseases, including asthma/allergy (182), myasthenia gravis (183), MS (184), and Alzheimer’s disease (185, 186). Systematic analysis of let-7 family members revealed that overexpression of let-7c inhibited M1 polarization and promoted M2 polarization through the regulation of CEBP-δ expression (187). Though the authors do not report the effect of let-7c on SOCS expression, their observations are reminiscent of studies carried out in SOCS3^−/−^ macrophages discussed in the section above. The discovery of new let-7 family members and targets demonstrates there is a great deal that remains to be understood about this miRNA family in regulating SOCS expression and macrophage biology. Furthermore, these findings highlight the need to re-evaluate earlier results in order to better understand which family member is being described.

In addition to their ability to directly regulate SOCS expression in macrophages, miRNAs can be shuttled between cells via exosomes to modify SOCS expression. Exosomes are lipid-bilayer-enclosed vesicles that carry cellular proteins, mRNA, and miRNAs (188, 189). The membrane proteins appear in the same orientation as on the cell membrane, owing to two invaginations, one at the surface of the plasma membrane, during the formation of the endosome, and the second by the inward budding of the endosomal membrane (188). Exosomes can be taken up by virtually every cell in the body (189, 190). In the context of pulmonary inflammation, SOCS expression is critical to preventing inflammatory cell influx and pathology. Exosomes secreted from allergic epithelial cells have been shown to influence chemokine sensitivity, trafficking, and signaling. Levänen et al. also identified altered miRNA expression in exosomes from the BAL fluid of allergic patients (191). They went on to identify several miRNAs known to regulate SOCS1 and SOCS3 expression. Bourdonnay et al. (192) reported that alveolar macrophages are capable of secreting microparticle vesicles laden with SOCS3 to epithelial cells, which were subsequently taken up. Delivery of SOCS3 to epithelial cells attenuated inflammation by blunting STAT signaling in response to IFN-γ or IL-6 (192). Taken together, these findings suggest not only that alveolar macrophages can influence epithelial cell expression of SOCS proteins, but that the reverse is also possible, potentially through exosome-mediated delivery of miRNA. These same mechanisms may allow DCs to influence T cell polarization. This novel pathway of SOCS delivery could influence cellular responsiveness and could also prove potentially useful in a therapeutic setting.

## Targeting miRNAs as Therapeutic Strategy

Modulating miRNA expression levels to fine-tune target gene expression is an attractive approach to enhancing inflammation in the case of vaccine immune responses, which can also be used to dampen inflammation in the case of autoimmune diseases. The effect of mir-155 on SOCS1 expression has been studied extensively and *in vitro* experiments suggest that delivering anti-mir or mir-155 neutralizing nucleotides can effectively increase SOCS1 expression and attenuate inflammation (193–197). Targeted delivery of anti-mirs to specific cell types such as T cells, macrophages, and microglia has clearly demonstrated the potential of this technology in modulating inflammation. More importantly, systemic delivery of antisense peptide nucleic acids has been effective in rodent models of MS (193, 198, 199), Alzheimer’s disease (200, 201), ALS (202), and ischemic stroke (202). These findings are significant in that systemic delivery altered mir-155 targets throughout the body, including microglia in the brain. This approach, coupled with advancements in polymer delivery technology and the efficiency and specificity of siRNA delivery, suggests that targeting SOCS-modifying miRNA may be feasible as a stand-alone or adjunct therapy for treating inflammatory disease. In addition, delivery of drug- or biological agent-conjugated poly(methyl methacrylate) nanoparticles (203) directly to effectively targets macrophages and microglia, making it possible to specifically target microglial SOCS expression in the CNS. Similarly, these nanoparticle delivery modes may be an option to target SOCS expression at the mucosal surface. For patients with asthma, globally targeting miRNAs known to promote Th2 responses or regulators of cytokine signaling, including M2 signaling, may be a safer approach to restoring immune regulation than targeting the systemic compartment, which may lead to undesired off-target effects.

## Therapeutic Delivery of SOCS Peptides and Full-Length Proteins

A large emphasis has been placed on development of short, cell-penetrative peptides that mimic the activity of the SOCS KIR. The KIR domain of the SOCS proteins is a 12-amino acid sequence that binds the activation loop of JAK2, inhibiting its kinase function. The SOCS1-KIR peptide was shown to inhibit STAT1α activation in response to IFN-γ in mouse macrophages (204). Additionally, a 16-amino acid SOCS1-KIR peptide delivered to EAE mice greatly diminished disease scores compared to those of animals that received control peptide (205). The improved symptom scores were shown to result from inhibition of Th1/Th17 development (IFN-γ production and inhibited IL-23 signaling) and reduced lymphocyte infiltration of the CNS. A shorter SOCS1 mimetic peptide, Tkip (tyrosine kinase inhibitory peptide), designed based on complementarity to the JAK2 activation loop, also reduced inflammatory responses in the same relapsing-remitting EAE model (206). Doti et al. (207) used an alanine scanning approach to determine key KIR residues involved in JAK2 binding. Their experiments resulted in the development of a shorter, 10-amino acid SOCS1 KIR, named “New KIR,” which has greater affinity for JAK2. A screen of peptides generated by random incorporation of unnatural amino acids into the “new KIR” allowed for selection of another peptide called PS-5, which has increased affinity for JAK2 and enhanced protease stability. PS-5 blocked the IFN-γ-induced STAT1/IRF-1 cascade that typically induces expression of integrins, chemokines, and MHCs from keratinocytes and recruits and activates immune cells in the skin (208). The authors demonstrated these capabilities both in a human keratinocyte cell line and in skin explants from human donors. Based on these *in vitro* results, the PS-5 peptide holds great therapeutic potential for treatment of psoriasis, although its activity *in vivo* remains to be determined. Another condition that may be a candidate for SOCS1-mimetic peptide intervention in macrophages is insulin resistance. Dampening proinflammatory responses of the macrophages in adipose fat may prevent the inflammatory conditions that lead to insulin resistance (209). Because overexpressed SOCS1 leads to insulin insensitivity through proteasomal degradation of IR and IRS-2 (10, 210, 211), extensive *in vivo* analysis of these novel SOCS1 KIR peptides would be necessary before consideration of human therapeutic use.

Delivery of full-length SOCS proteins or SOCS overexpression may be an additional mechanism by which to influence polarization of macrophages and/or DCs. Disease processes that are dependent on the polarization and activity of macrophages, such as plaque formation in atherosclerosis, may be good targets for a SOCS-based therapy. Adenovirally overexpressed SOCS1 (Ad-S1) reduced the accumulation of lipids and macrophages in the plaques of ApoE-deficient mice fed a high-fat diet (212). Interestingly, the blood of the Ad-S1-treated animals contained fewer Ly6C^hi^ monocytes and more Ly6C^lo^ monocytes than did that of controls, suggesting diminished systemic inflammation with SOCS1 treatment. Exciting new findings showed that alveolar macrophages in the lung secrete exosomes containing SOCS1 and SOCS3 proteins to control airway epithelial responses to cytokines (192). The secretory process becomes dysregulated in the alveolar macrophages of smokers and likely results in uncontrolled inflammation. Whether macrophages are also recipients of exosomally delivered SOCS proteins was not investigated, although exosomal or microparticle uptake by macrophages is likely, given their role as major “professional” phagocytes. Exploiting macrophage uptake processes may be a useful way to deliver therapeutic SOCS proteins or peptides to modify macrophage polarization.

Although enhancing the activity of SOCS proteins to suppress inflammatory responses would be advantageous, in some situations, enhancing immune responses is desirable, such as for clearing viral or bacterial pathogens and eliciting robust vaccine responses. In these cases, the suppressive activity of the SOCS proteins is unfavorable, and approaches are needed to reduce the expression or function of SOCS proteins. For example, to augment anti-tumor vaccine responses, Zhang et al. (213) tried silencing SOCS1 expression in DCs to enhance TLR/NF-κB signaling *in vitro*. They nucleofected DCs with a plasmid that encoded an shRNA to SOCS1; overexpressed MAGE3, a tumor antigen; and overexpressed HMGB1 to stimulate NF-κB signaling. SOCS1 expression was efficiently downregulated in the nucleofected DCs, and the cells were more potent at inducing Th1-polarization. In a similar approach, Zhu et al. (214) downregulated SOCS1 in DCs *in vitro* by using an adenoviral vector that expressed shRNA to SOCS1 (Ad-shRNA-SOCS1). Their study went further and demonstrated that vaccination with Ad-shRNA-SOCS1 was able to shrink tumors and increase survival in tumor-injected mice.

Peptide antagonists have also proved useful in inhibiting the activity of the SOCS proteins. SOCS1 antagonist peptides are essentially the amino acid sequence of the activation loop of JAK2. The SOCS1 antagonist pJAK2 peptide (also called lipo-pJAK2) is a tyrosine-phosphorylated, 13-amino acid, palmitoylated peptide that blocks SOCS1 activity and enhances STAT3 activation in IL-6-stimulated human LNCaP cells (204). In very promising studies carried out in human monocyte-derived DCs, the pJAK2 peptide enhanced the ability of human DCs to activate cytotoxicity of tumor-specific human CD8 + CTLs (215). Whether these anti-tumor effects can be recapitulated *in vivo* remains to be determined. Enhancing proinflammatory cytokine production with lipo-pJAK2 has beneficial antiviral effects. For example, mice that received intraperitoneal lipo-pJAK2 were protected from a lethal dose of vaccinia virus (216). In the same report, lipo-pJAK2 also afforded 60–80% protection against encephalomyocarditis virus.

The efficacy of SOCS-based peptides and proteins will depend on their uptake, stability, and ability to specifically target macrophages and DCs. To this end, a cell-penetrative SOCS3 (CP-SOCS3) was engineered to include hydrophobic amino acid sequences for translocating the cell membrane (125). Fluorescently labeled CP-SOCS3 was taken up efficiently by blood leukocytes and lymphocytes and was retained for up to 24 h. CP-SOCS3 was able to block inflammation in mice *in vivo*, and deletion of the SOCS box of CP-SOCS3 significantly increased the half-life of the protein (217). Using non-natural amino acids as part of the peptide, SOCS1 KIR sequence provided enhanced stability against proteases in the case of PS-5 (207). The SOCS proteins are attractive therapeutic targets because they are potent regulators of macrophage and DC polarization, but care must be exercised in their utilization as regulators of inflammation for therapeutic benefit. Over- or under-regulation of the pro- or anti-inflammatory responses of macrophages and DCs could have detrimental effects. Nonetheless, understanding the biology of these regulatory proteins in macrophages, microglia, and DCs may lead to promising novel interventions for a variety of immune-mediated diseases.

## Concluding Remarks

The SOCS family of proteins plays a pivotal role in macrophage and DC biology. Of the eight family members, it is clear that SOCS1 and SOCS3 are key players and have discrete, non-redundant roles in regulating macrophage and DC polarization and cytokine signaling. These differences also have important implications in host resistance to infection and regulation of immune responses. Targeting select SOCS family members in macrophages has immense therapeutic potential. New technologies and therapies aimed specifically at modulating SOCS expression may be an effective way to treat disease.

## Author Contributions

SM and NH co-wrote the manuscript.

## Conflict of Interest Statement

The authors declare that the research was conducted in the absence of any commercial or financial relationships that could be construed as a potential conflict of interest.
